# Conversion of Sleeve Gastrectomy to Roux-en-Y Gastric Bypass to Enhance Weight Loss: Single Enterprise Mid-Term Outcomes and Literature Review

**DOI:** 10.1089/bari.2021.0096

**Published:** 2022-12-14

**Authors:** Gabriel Diaz Del Gobbo, Nada Mahmoud, Juan S. Barajas-Gamboa, Michael Klingler, Paola Barrios, Carlos Abril, Javed Raza, Ali Aminian, Raul J. Rosenthal, Ricard Corcelles, Matthew D. Kroh

**Affiliations:** ^1^ Department of General Surgery, Digestive Disease Institute, Cleveland Clinic Abu Dhabi, Abu Dhabi, United Arab Emirates.; ^2^ Cleveland Clinic Lerner College of Medicine, Case Western Reserve University, Cleveland, Ohio, USA.; ^3^ Department of General Surgery, Bariatric and Metabolic Institute, Cleveland Clinic, Cleveland, Ohio, USA.; ^4^ Department of General Surgery, Bariatric and Metabolic Institute, Cleveland Clinic Florida, Weston, Florida, USA.

**Keywords:** sleeve, revision, Roux-en-Y gastric bypass, conversion, non-responders, weight loss

## Abstract

**Background:**

Suboptimal weight loss (SWL) occurs up to 30% after sleeve gastrectomy (SG). Conversion to Roux-en-Y gastric bypass (cRYGB) has shown heterogeneous results in terms of additional weight loss and resolution of weight-related comorbidities. We aim to evaluate mid-term outcomes of cRYGB specifically for SWL after SG.

**Methods:**

All patients who underwent cRYGB for SWL from April 2010 to June 2019 from prospective registries at three affiliated tertiary care centers were retrospectively reviewed. Patients who underwent revision or conversion for complications were excluded. Mixed-effects and polynomial regression models were used to evaluate weight loss results after conversion.

**Results:**

Thirty-two patients underwent cRYGB from SG. About 68.7% were women with mean age of 46.6 years. Mean body mass index (BMI) before SG was 55.3 kg/m^2^. Before conversion, mean BMI was 44.5 kg/m^2^ with 17.3% total weight loss (TWL). All procedures were completed laparoscopically in a median surgical time of 183 min. Three major complications occurred (9.3%), one gastrojejunal (GJ) leak and two reoperations. Four cases (12.5%) of GJ stenosis were diagnosed. No mortality was registered. Mean follow-up time was 24 months and patients had 36 kg/m^2^ mean BMI, 17.4% TWL, 27.2% had BMI >35 kg/m^2^.

**Conclusions:**

cRYGB after SG for SWL showed good mid-term results, better than those reported in literature.

## Introduction

Sleeve gastrectomy (SG) was initially proposed as staged procedure for high-risk patients with super obesity. With time, it gained recognition as a stand-alone intervention to achieve weight loss and resolution of comorbidities.^1^ This procedure has been rapidly adopted by the surgical community and currently accounts 46% of all bariatric operations performed globally.^2,3^

SG has favorable long-term outcomes by achieving 50–65% mean excess weight loss (EWL) at 5 years.^4–8^ However, 28% of patients might experience suboptimal weight loss (SWL) at a longer follow-up, and almost a quarter of them will seek a second surgery to achieve successful weight reduction.^9,10^

Conversion to Roux-en-Y gastric bypass (cRYGB) is also commonly performed for correction of complications from the initial SG. Frequently this includes leaks, stenosis, torsion, hiatal hernia, and gastroesophageal reflux disease (GERD). cRYGB has been shown to improve obstructive symptoms after SL.^11–14^ Several groups have reported up to 75–100% symptom resolution.^12,15,16^

There is no validated protocol or consensus on which is the most effective surgical approach for SWL. At present, cRYGB and biliopancreatic diversion with duodenal switch (BPD/DS) are the most common revisional procedures.^17,18^ Conversion to BPD/DS can be technically challenging with increased risk of postoperative complications and long-term nutritional consequences. Gastric bypass with long biliopancreatic limb provides better weight loss than Roux-en-Y gastric bypass (RYGB) at the cost of nutritional deficiencies.^19,20^ Results from newer surgical procedures such as the one anastomosis gastric bypass (OAGB) and the single-anastomosis duodenoileal bypass with sleeve gastrectomy (SADI-S) suggest that they might be good treatment options. Nonetheless, OAGB has increased rates of bile reflux and ulcers, and like BPD-DS although maybe not as frequent, SADI-S may induce protein-calorie malnutrition.^21–23^

cRYGB has been previously demonstrated to be safe and effective for the treatment of SWL after SG.^4^ However, definitions for successful SWL and metrics implemented for follow-up analyses are heterogeneous across studies and limit conclusions. The aim of this study was to evaluate mid-term outcomes of patients undergoing cRYGB for SWL after SG from three separate institutions of a single hospital system, and to conduct a literature review by means of harmonized metrics.

## Materials and Methods

We conducted a retrospective review of prospective bariatric surgery registries across the three major teaching institutions of the Cleveland Clinic enterprise. Research and ethics committee approval was obtained separately at each institution. Data from patients undergoing cRYGB from SG at the three separates hospitals (two in the United States, one in United Arab Emirates) were collected from April 2010 to June 2019. Only those patients who underwent conversion SG to cRYGB for insufficient weight loss or weight regain as the main indication were included in the analysis. We excluded those cases who underwent conversion for mechanical complications such as stenosis, torsion, or hiatal hernia with obstructive symptoms. SWL was defined as EWL <50% or body mass index (BMI) ≥35 kg/m^2^ at 2 years from primary procedure. Patients younger than 18 years old, those who had cRYGB for indications other than SWL, or patients who had a conversion to a different procedure other than standard RYGB were excluded. Weight values were extracted for any hospital encounter at 1–3, 6–12, 24–36, and 48–60 months postconversion.

### Preoperative evaluation and surgical approach

All patients diagnosed with SWL after SG underwent a multidisciplinary workup before revision, as is standard practice at all three institutions. Most patients underwent an upper endoscopy and contrast-enhanced upper gastrointestinal series and were evaluated by a team including at a minimum, the operating surgeon, dietician, psychologist, and internal medicine/obesity medicine specialist. Additional tests, such as esophageal manometry, pH probe placement, and referral to other specialists were carried out at the team's discretion and patient's needs. Given that the primary operations as well as the reoperations were carried out across three different sites, there was variability in the techniques used. In general, adhesiolysis was performed to free the stomach from all attachments and to identify the hiatus. A 30–50 mL pouch was constructed. The jejunum was divided at 40–60 cm from the ligament of Treitz. A 100–180 cm alimentary limb was created. The gastrojejunal (GJ) anastomosis was fashioned according to surgeon's preference using either handsewn, linear, or circular-stapled technique.

### Calculations and statistics

Data were collected in Microsoft^®^ Excel 2019 datasheet and analyzed using Microsoft R Open 3.5.3. Descriptive statistics were calculated as mean ± standard deviation or median and interquartile range (IQR) depending on distribution characteristics. Categorical variables were described in terms of counts and percent. Reporting parameters were implemented according to the American Society of Metabolic and Bariatric Surgery (ASMBS) outcome reporting standards.^24^

To assess changes in weight, BMI, and %TWL over time, we utilized mixed-effects regression models. Specifically, repeated measures were nested in patients, and a patient-level random intercept term was included to account for the longitudinal data structure. Change trajectories were modeled using a fourth-degree polynomial at the repeated-measures level. Polynomial coefficients were allowed to nonrandomly vary as functions of baseline weight, BMI, or %TWL. Conceptually speaking, this is equivalent to allowing expected trajectories in outcome variables to vary depending on baseline characteristics. We note that mixed-effects regression models allow for any subject with at least one data point to contribute to the estimated trajectories. Thus, a subject is not required to have data at all time points to be included in our analyses. Mixed-effects regression models account for any missing data through Bayesian weighted estimates of trajectories that incorporate subject-specific reliabilities into the weighting scheme. Such methods have been shown to produce consistent and unbiased estimates of longitudinal effects in the presence of either missing completely at random or missing at random data.^25^

### Literature review

**FIG. 1.**
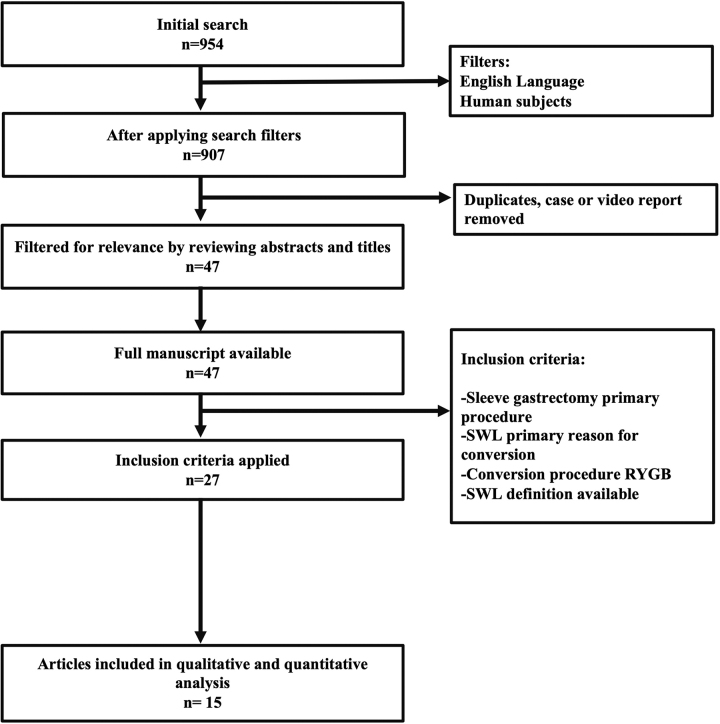
Flow diagram of article selection process. RYGB, Roux-en-Y gastric bypass; SWL, suboptimal weight loss.

A literature review was carried out by searching the PubMed, Scopus, JSTOR, SAGE journals, and Cochrane databases. Search terms included “sleeve gastrectomy,” “gastric sleeve,” “conversion,” “revision,” “bypass,” “Roux-en-Y,” “weight loss,” “insufficient,” “inadequate,” “weight regain,” and “weight loss failure” to find articles focusing on the conversion of SG to RYGB in the light of SWL postoperatively. G.D.D.G. and N.M. independently performed the literature search, results were cross-checked to achieve consensus, and J.S.B.-G. was involved in cases of discrepancy. Studies were included if they were in English, published up to February 2021, if SG was performed as a primary procedure, and had reported weight evolution at follow-up. Articles were excluded if there was no clear definition of SWL, if they were case or video reports, if the article was unavailable or if we were not able to extract results of conversion only for SWL in publications with combined data (GERD and mechanical complications). A total of 954 articles were initially identified, 939 were excluded from analysis, and 15 were deemed appropriate for analysis. Complete protocol for article selection is given in Figure 1. Metrics were extracted or calculated when possible using the following formulas: %EWL = [(preconversion weight) − (postoperative weight)]/[(preconversion weight) – (ideal weight)]^24^ or %TWL = [(preconversion weight) – (postoperative weight)]/[(preconversion weight)] × 100,^26^ to improve standardization and facilitate the literature appraisal. A meta-analysis was not carried out as we found great heterogeneity in the definition for SWL. The summary of results from these studies are given in Table 1.

**Table 1.** Available Results in Literature About Conversion to Roux-en-Y Gastric Bypass After Sleeve Only for Suboptimal Weight Loss

Study	Country	Year of publication	Definition of SWL	Time to conversion	Number of patients	Age (years)	Sex	BMI at primary (kg/m^2^)	BMI at conversion (kg/m^2^)	BMI at FU (kg/m^2^)	%TWL	%EWL
Dijkhorst *et al.*^23^	The Netherlands	2018	BMI >35 kg/m^2^	NA	45 patients	NA	NA	NA	42.5 ± 6	24 m: 39.5 ± 5.5	3 m: 5.9 ± 5.3% 6 m: 7.8 ± 6.8% 12 m: 8.9 ± 8.7% 24 m: 6.9 ± 11.3%	NA
Andalib *et al.*^41^	Canada	2020	EWL <50% ≥20% weight regain of the weight lost	34 m	33 patients	51	NA	49.5	40.1	12 m: 35	12 m: 10.1%	12 m: 27.6%
Malinka *et al.*^13^	Switzerland	2017	<50% EWL after 12–18 m	19.56 ± 12.16 m	32 patients	42.3 ± 11.5 m	22F 10M	49.4 ± 5.3	40.9 ± 5.6	36 m: 33.4 ± 5.9	36 m: 18.7%	36 m: 52 ± 26
Poghosyan *et al.*^27^	France	2016	EWL <50% at 2 years	32 (8–69) m	31 patients	NA	NA	53.3 ± 11.5	44.7 ± 9.8	6 m: 38.5 ± 8.8 12 m: 39.1 ± 9.1 18 m: 40.4 ± 9 24 m: 40.3 ± 9.5 36 m: 40.9 ± 8.5	6 m: 27.3 ± 8% 12 m: 25.9 ± 11% 18 m: 23.8 ± 9.9% 24 m: 23.8 ± 10% 36 m: 23.8 ± 14%	NA
Shimon *et al.*^29^	Israel	2018	BMI >35 kg/m^2^	47 ± 20 m	18 patients	43.94 ± 16.6	10F 8M	46.1 ± 5	40.5 ± 27	12 m: 32.3 ≥ 24 m: 31.9	12 m: 18.7 ± 10% ≥ 24 m: 21 ± 14%	NA
Rosa Marti-Fernandez^49^	Spain	2020	EWL <50% BMI >35 kg/m^2^	NA	18 patients	NA	NA	61.7 ± 8.5	45.6 ± 8.7	^*^Last FU: 39.8 ± 8.6	^*^Last FU: 7.4%	NA
Boru *et al.*^28^	Italy	2018	IWL: <50% at 12 m FU <20% loss of initial weight BMI ≥35 kg/m^2^ at last FU WR: 5–10 kg from nadir	NA	12 patients	NA	NA	NA	41.2 ± 6.3	6 m: 33.3 ± 4.6 12 m: 32.1 ± 4.8 24 m: 31.1 ± 5.5	NA	NA
Salman Al Sabah^50^	Kuwait	2016	EWL <50% at 1 year	NA	12 patients	34	10F 2M	52	48.3	3 m: 42 6 m: 39 12 m: 36	3 m: 12% 6 m: 17.6% 12 m: 24.8%	3 m: 26% 6 m: 29% 12 m: 61%
Homan *et al.*^21^	Netherlands	2014	IWL <50% EWL Weight regain >25% EWL regain	30 (9–56) m	11 patients	NA	NA	50 (40–59)	39 (36–48)	NA	^*^Last FU: 9%	34 m: 23% (49–84)
Parmar *et al.*^12^	United Kingdom	2017	EWL <50%	NA	11 patients	54 (36–70)	8F 3M	53.1 (42.3–66.2)	43.3 (34.6–54.4)	6 m: 38.2 (31–46.1) 12 m: 39.9 (30.4–47.6) 24 m: 40.8 (32.3–48.1)	6 m: 11.23% 12 m: 7.43% 24 m: 6.22%	6 m: 49.9% (32.8–68.9) 12 m: 49.5% (33.7–72.5) 24 m: 46% (32.3–57.7)
Landreneau *et al.*^40^	United States	2018	EWL <50%	19 m	11 patients	NA	NA	NA	48.6 (39.3–50.2)	1 m: 46.3 12 m: 40.7	12 m: 16.1% (8.2–20.2)	12 m: 32.7% (22.8–41.7)
Idan Carmeli^51^	Israel	2015	BMI >35 kg/m^2^	36 ± 17 m	10 patients	NA	7F 3M	44.5 ± 5	39.8 ± 5.7	16 m: 30.2 ± 4.8	16 m: 24.29%	12 m: 77 ± 110% 16 m: 66.6 ± 33.9%
Thomas Gautier^52^	France	2012	Stable weight for 6 m with BMI 40 kg/m^2^ or above	24.3 m	9 patients	36.8	NA	58.2	43.7	15 m: 38.1	NA	15 m: 59%
Abraham Abdemur^53^	United States	2015	EWL <50%	NA	7 patients	NA	NA	NA	NA	NA	NA	9 m: 47%
Amiki *et al.*^14^	Japan	2020	EWL <50%	24 (12–180) m	2 patients	39.5	1F 1M	53.1	49	48 m: 40.3	48 m: 18.3%	48 m: 38.5%

^*^ Not possible to identify the exact time of FU.

%EWL, percentage of excess of weight loss; %TWL, percentage of total weight loss; BMI, body mass index; F, female; FU, follow-up; IWL, inadequate weight loss; M, male; NA, not available; SWL, suboptimal weight loss; WR, weight regain.

## Results

### Demographics

Thirty-two patients underwent cRYGB for SWL as the main indication during the study period. Twenty-two (68.7%) were women with a mean age of 46.6 ± 13.4 years. Mean BMI before sleeve was 55.3 ± 10 kg/m^2^ with 92% of data retention. Half of the index SG (18/32) were performed at one of our hospitals with an interval time to conversion of 51 ± 24.2 months. At the time of cRYGB, the mean BMI was 44.5 ± 7 kg/m^2^, which corresponded to 17.3 ± 11.9% TWL and 29 ± 17.7% EWL. Ninety percent had a comorbidity at the time of conversion with an average of 2.2 per patient: GERD 43.7%, obstructive sleep apnea (OSA) 40.6%, dyslipidemia 37.5%, and type 2 diabetes mellitus (T2DM) 31.2%. Although GERD was significantly present, the primary indication for conversion in this series was SWL. Patients who presented with GERD as their major complaint, had evidence of erosive esophagitis or complications of GERD including strictures, and those with symptoms refractory to medical treatment were excluded. About 18.5% of patients were on weight loss medications at the time of conversion. Demographic data are summarized in Table 2.

**Table 2.** Descriptive Data

	*n* (%)
Sex (*N* = 32)	
Female	22 (68.7)
Male	10 (31.2)
Age (years)	46.6 ± 13.4
Weight at primary (kg)	150 ± 29.7
BMI at primary (kg/m^2^)	55.3 ± 10
Weight at conversion (kg)	121.2 ± 22.7
BMI at conversion (kg/m^2^)	44.5 ± 7
%EWL at conversion	29 ± 17.7
%TWL at conversion	17.3 ± 11.9
Time to conversion (months)	51 ± 24.2
Perioperative weight loss medications	6 (18.7)
Comorbidities	29 (90.6)
T2DM	10 (31.2)
HTN	9 (28)
Dyslipidemia	12 (37.5)
Fatty liver	4 (12.5)
GERD	14 (43.7)
OSA	13 (40.6)
Depression	5 (15.6)

GERD, gastroesophageal reflux disease; HTN, hypertension; OSA, obstructive sleep apnea; T2DM, type 2 diabetes mellitus.

### Overall operative outcomes and complications

All procedures were completed laparoscopically, in a median time of 183 (IQR = 143–207) minutes. No conversion to open procedures or intraoperative complications was experienced. Remnant gastrostomy tubes were used in 15.6% of patients, and drains were placed in 40.6% of cases. Both remnant gastrostomy and drain placement were more common in our early experience and are not part of our current practice at any site. Median postoperative stay was 3 (IQR = 2–4) days.

Postoperative complications were registered in 10 patients, 3 were major and seven minors. During the early postoperative period (<30 days) eight complications occurred. GJ anastomotic stenosis developed in three patients and all were successfully treated with endoscopic dilation with controlled radial expansion balloon. One of these patients suffered a perforation during dilation and underwent surgical revision. The rest of complications were one patient with contained GJ leak that was treated nonoperatively, one bowel obstruction in a patient with significant adhesions secondary to previous surgeries that required a reoperation. Two blocked gastrostomy tubes and one urinary tract infection also occurred. At >30 days, one patient had a GJ anastomotic stenosis requiring dilation, and one patient developed a jejunojejunostomy intussusception that was managed nonoperatively.

Five re-admissions (15.6%) were documented at ≤30 postoperative days, the majority (3/5) were owing to GJ stenosis. No mortality was seen during the early postoperative period, two deaths occurred during the follow-up period, both unrelated to the revisional surgery.

### Weight-related outcomes

Mean follow-up was 24 ± 18 months. Weight data evolution was available for 77.7% (21/27) at 1 year, 59% (12/22) at 2 years, 71.4% (10/14) at 3 years, 70% (7/10) at 4 years, 37.5% (3/8) at 5 years. Results from the mixed-effects and polynomial regression models showed weight loss nadir at 1-year postconversion with a mean BMI of 34.8 ± 6.9 kg/m^2^ and an additional 20 ± 4.4% TWL (Table 3).

**Table 3.** Weight Evolution Based on Mathematical Model After Conversion to Gastric Bypass

	Before cRYGB	1 month	3 months	6 months	12 months	24 months	36 months	48 months	60 months
Weight (kg)	121.2	114.3	107.6	100.6	94.6	97.7	99.6	94.1	97.3
BMI (kg/m^2^)	44.5	42	39.6	37	34.8	36	37	36.2	38.6
%TWL	17.3	3.4	9.1	15	20	17.4	15.9	20.5	17.8

cRYGB, conversion Roux-en-Y gastric bypass.

**FIG. 2.**
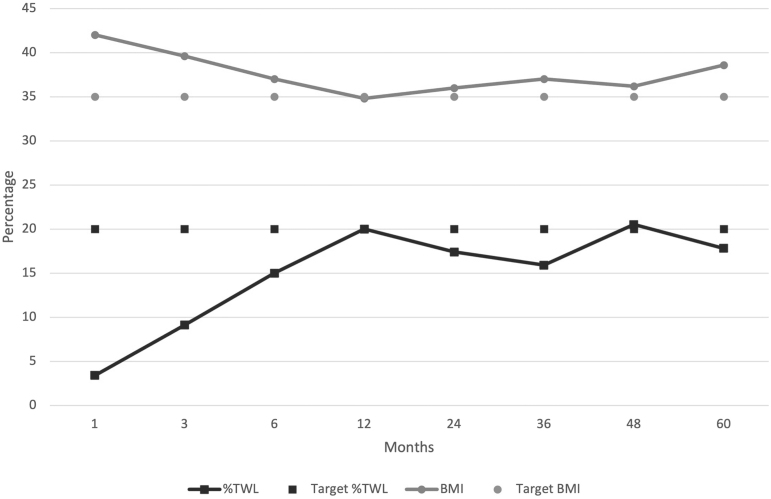
BMI and %TWL evolution after conversion to RYGB based on mathematical model. %TWL, percentage of total weight loss; BMI, body mass index.

At 2 years, patients had a mean BMI of 36 kg/m^2^ and 17.4% TWL. Twenty-seven percent of cases (6/22) had BMI >35 kg/m^2^ and 31.8% (7/22) had <20% TWL. Patients with 150 cm Roux limb had better weight loss than those with 100 cm (mean BMI 35 vs. 37.8 kg/m^2^ and %TWL 18.8 vs. 15.2). At 4 years the patients had mean BMI 36.2 ± 6.9 kg/m^2^ and 20.5 ± 4.5% TWL (Figure 2).

## Discussion

The objective of this study was to evaluate outcomes of cRYBG in patients who experienced SWL after SG. In general, the most common indication for revision after SG is refractory GERD or other mechanical complications from the primary operation, including hiatal hernia, stenosis, or torsion. However, SWL is becoming more prevalent as the volume of patients undergoing SG has dramatically increased in the past decade. There are few studies evaluating longer term outcomes of cRYBG after SG for SWL. Most of the existing data do not clearly differentiate between conversions for complications versus SWL alone.

Literature review showed limited efficacy and high variability. After analyzing the publications that meet criteria for inclusion in our study, most patients did not reach successful weight loss at 2 years post-cRYGB. %TWL ranges from 7% to 24% and BMI from 39.5 to 41 kg/m^2^.^12,23,27,28^ Only two studies showed TWL ≥20% at 2 years from conversion; these series showed 31 and 40 kg/m^2^ mean BMI.^27,29^ Similar conflicting results exist at 3 years of follow-up, with cRYGB resulting in 18–24% TWL and BMI 33–41 kg/m^2^.^13,27^ According to the mixed-effect and polynomial regression models performed in our study, 2 years after cRYGB our patients had better outcomes than those available in the literature. Our series showed a mean BMI of 36 kg/m^2^ corresponding to an additional 17.4% TWL. This appeared to be sustained at a longer follow-up too. Our results must be carefully interpreted as our mean follow-up is 24 months with few patients at 4 years postconversion (Table 3).

At 2 years post-cRYGB, we found that 27.2% of our patients had BMI >35 kg/m^2^ and 31.8% had <20% TWL. These outcomes do not necessarily mean that this intervention lacks metabolic benefit. It is reported that experiencing SWL after a primary bariatric operation may still result in 23% of diabetes remission and significant decrease in medication requirements. Up to 60% of these patients can reach the levels of blood pressure control and low-density lipoprotein levels recommended by the American Diabetes Association.^30^ A more recent study showed beneficial impact in mortality, coronary artery events, cerebrovascular events, heart failure, atrial fibrillation, and nephropathy with even 10% of total body weight loss after surgery.^31^

In general, available data regarding cRYGB for SWL alone after SG is limited and heterogeneous. Most existing studies have a small sample size ranging from 2 to 45 patients, and are retrospective single institution in nature. There is short-term follow-up and significant variability in the indications for cRYGB rather than weight regain or inadequate weight loss in isolation. Two studies with 31 and 32 patients, respectively, have results of 3 years postconversion.^13,27^ One of the latest series includes two patients with 48 months of follow-up.^14^ Our study is among the largest cohorts analyzing outcomes for SWL, having 32 patients with ≥60% data retention of weight metrics during 4 postoperative years and a mathematical model was implemented to accurately compensate missing values.

Weight regain is associated with relapse of comorbidities,^32–34^ which is poorly discussed in the literature. We found a significant metabolic burden in our patients where 90.6% had an obesity-related comorbidity such as OSA (40%), dyslipidemia (37%), T2DM (31%), hypertension (28%), and fatty liver (12%) after SG. Medical therapies have been used to treat SWL, with adjuvant weight loss medications showing limited potency achieving %TWL 2–5.^35,36^ In this series, 18.7% of the patients were taking a weight loss medication at the time of conversion.

The ASMBS published in 2015 a series of recommendations for standardized outcome reporting in bariatric and metabolic research.^24^ However, a wide range of definitions have been used for SWL, and we found seven different definitions in the literature reviewed. Such variability challenges the comparison across studies and limits the ability to perform constructive data evaluation. At present, there is no unique parameter to measure SWL in primary bariatric surgical operations, but the most commonly reported metric is EWL <50%. It is known that the most adequate metric to identify poor responders is TWL <20%.^24,37^ In efforts to reduce the outcomes disparities and to enable a robust evaluation, we calculated both %EWL or %TWL for all groups when possible (Table 1).

Revisional bariatric surgery can be associated with increased postoperative complications ranging from 15% to 40%.^11,21,38,39^ To safely recommend conversion for weight regain or accruement of weight-related comorbidities, it is appropriate to evaluate the risks and benefits of the intervention. As such, it is important to examine the complication profile of patients who underwent cRYGB for SWL apart from those who underwent conversion for mechanical problems. Having higher BMI at conversion in cases of SWL potentially provides increased technical challenges, including less visible surgical fields with reduced intra-abdominal cavity space, substantial port torque, enlarged liver, and increased intra-abdominal fat. From the publications that we reviewed, only two articles reported the postoperative complications only for the group with SWL, having an incidence of 29.2–45.5%.^40,41^

Significance of the time interval between SG and then cRYGB is not yet well established and is likely variable between patients. A two-stage approach has previously been described for patients with very high BMIs or challenging anatomic situations. The intention is to perform the second stage under more favorable anatomical conditions. In one study, a 40-month gap was reported in a group of 10 patients with a similar rate of postoperative complications.^42^ This time frame was recognized in 62% of the publications included that we reviewed, with a mean of 29 months. In our series, the interval to conversion was longer (52 months).

Until recently, the conversion of SG into a BPD/DS has been the most effective strategy to achieve successful weight loss. It is known that it can be technically demanding, patients may develop protein–calorie malnutrition, and adverse gastrointestinal symptoms.^29,43^ Long biliopancreatic limb gastric bypass has shown better weight loss outcomes in comparison with standard RYGB, but at the cost of higher rate of nutritional deficiencies.^19,20^ OAGB is an effective revisional procedure with BMI reduction from 43 to 35 kg/m^2^, representing 21% TWL at 1-year postconversion.^44^ Sustained weight loss has also been reported at a long term.^45^ However, complication rates occur not infrequently, such as 6.1–26% of bile reflux and 2.4–6.1% of anastomotic ulcers.^44,45^

SADI-S has recently emerged as reasonable option for conversion owing to SWL with favorable weight loss outcomes and acceptable perioperative morbidity.^23,46–48^ In a report, it seemed to be more effective than cRYGB for SWL at 2 years postconversion (%TWL 26.4 ± 10 vs. 7 ± 11) with similar rate of vitamin deficiencies (64% vs. 62%).^23^ These promising results come from retrospectives studies with modest cohorts. More impactful data may drive our future practices.

Shortcomings of the study include that this is a highly select cohort of patients and technical details of the index operation were missing in half of the cases as they were performed at other institution. Weaknesses of this study also include the inherent limitations of registry data, the relatively small number of patients enrolled, and lack of standardization in technique between our centers and the reported literature. Our study is among the largest series documenting cRYGB for SWL alone. The strengths of this publication results from a high rate of data retention, a mathematical model to estimate missing values, and a literature review with homogeneous metrics. To our knowledge, no other publication addressing our objective has implemented these methodologies. In addition, the results may be more broadly generalizable with the inclusion of three distinct patient populations.

## Conclusion

Performing cRYGB after SG for SWL alone may have better mid-term results than those reported in the literature. Published data addressing this issue is heterogeneous with a considerable amount of overlapping data for complications and weight gain both. Robust studies with clear definitions should be carried to identify the proper strategy to treat our patients.
